# Energy dissipation in flows through curved spaces

**DOI:** 10.1038/srep42350

**Published:** 2017-02-14

**Authors:** J.-D. Debus, M. Mendoza, S. Succi, H. J. Herrmann

**Affiliations:** 1 ETH Zürich, Computational Physics for Engineering Materials, Institute for Building Materials, Wolfgang-Pauli-Str. 27, HIT, CH-8093 Zürich, Switzerland; 2 Instituto per le Applicazioni del Calcolo C.N.R., Via dei Taurini, 19 00185, Rome, Italy

## Abstract

Fluid dynamics in intrinsically curved geometries is encountered in many physical systems in nature, ranging from microscopic bio-membranes all the way up to general relativity at cosmological scales. Despite the diversity of applications, all of these systems share a common feature: the free motion of particles is affected by inertial forces originating from the curvature of the embedding space. Here we reveal a fundamental process underlying fluid dynamics in curved spaces: the free motion of fluids, in the complete absence of solid walls or obstacles, exhibits loss of energy due exclusively to the intrinsic curvature of space. We find that local sources of curvature generate viscous stresses as a result of the inertial forces. The curvature- induced viscous forces are shown to cause hitherto unnoticed and yet appreciable energy dissipation, which might play a significant role for a variety of physical systems involving fluid dynamics in curved spaces.

In many physical systems, the collective motion of particles constitutes a viscous flow, satisfying the laws of hydrodynamics. When the particles are confined to a surface, the flow forms a two-dimensional liquid film, which can be described effectively by the two-dimensional Navier-Stokes equations. In general, liquid films can be arbitrarily curved, which introduces interesting new effects originating from the interaction between the flow and the curvature of the film. An important example from microbiology are lipid bilayer membranes, forming the envelope of most of the cell components. Lipid bilayers possess hydrodynamic properties such as diffusion and viscosity, as it has been confirmed by various experiments^1^,^2^,^3^,^4^. Thus, lipid bilayers are commonly modeled as a two-dimensional viscous fluid moving on a curved surface^5^,^6^ according to the Navier-Stokes equations for manifolds^7^. Further examples of two-dimensional fluids on curved surfaces are liquid interfaces, such as interfaces between foam bubbles and molecular films around emulsions or aerosol droplets. Mathematically, these interfaces can be approximated by the two-dimensional Navier-Stokes equations on a sphere^8^,^9^. Furthermore, an attractive field of application is graphene, a two-dimensional gapless semiconductor, which has gained increasing popularity during the past decades due to its extraordinary properties. Recently, it has been shown that in a certain temperature range, the electrons in graphene behave collectively as a viscous fluid^10^, governed by the hydrodynamics equations^11^,^12^. A daily-life application of flow on curved surfaces are soap films, which have been widely used to study classical hydrodynamic phenomena in two dimensions^13^,^14^. Because of their negligible thickness, soap films follow the two-dimensional Navier-Stokes equations for small Mach numbers^15^, since, at leading order, variations in the film thickness can be neglected. In general, though, interactions with the surrounding air, such as Ekman friction^16^ or other interfacial effects^17^,^18^ contribute additional corrections to the hydrodynamic equations. Finally, on larger scales, the Earth’s atmosphere and the photosphere of the sun can also be modeled by hydrodynamic models on two-dimensional spheres, as discussed for example in refs 19 and 20.

While flows on curved surfaces appear in many fields of application, numerical models are often restricted to simplified geometries (e.g. spheres) or to fully incompressible fluids^21^. In this paper, we study flows on surfaces with a general curvature by using a lattice Boltzmann method for manifolds^22^,^23^,^24^. As an illustration, Fig. 1 shows our numerical simulation of a curved 2D fluid film (e.g. a soap film), flowing downwards between two wires. As one can appreciate, the vorticity field is amplified next to the center of the curvature bump, which induces velocity gradients and shear within the fluid flow. In the following section we study this effect in more detail and show that spatial curvature itself generates viscous forces, leading to curvature-induced energy dissipation in viscous flows.

## Results

### Energy dissipation in flows past a spatial deformation

In order to gain a deeper understanding of the underlying process, we consider a smooth curvature source, described by the metric tensor *g*_*ij*_ = (1 + *δ*_g_)*δ*_*ij*_ with a Gaussian perturbation *δ*_g_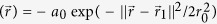, where 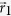 denotes the center position, *a*_0_ the amplitude and *r*_0_ the width of the perturbation. The corresponding curvature field can be quantified by the Ricci curvature scalar, as depicted in Fig. 2(a). As one can see, the positive curvature field causes an attractive inertial force field, which is driving the particles along the geodesic lines, the lines of constant kinetic energy in curved space. The simulation of the fluid is based on the two-dimensional covariant Navier-Stokes equations^25^,


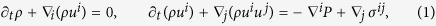


characterizing the time evolution of the fluid density *ρ* and velocity *u* on a 2D curved surface, equipped with a metric tensor *g*_*ij*_ and a covariant derivative ∇. Here, *P* denotes the hydrostatic pressure and *σ*^*ij*^ the viscous stress tensor, given by


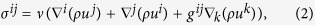


where *v* denotes the kinematic viscosity. Interestingly, we find that the flow converges to a stationary equilibrium state after sufficient time, even though we consider an open system where the fluid is continuously driven by a constant pressure drop. This effect contradicts the behavior of flows in flat space, which, in the absence of solid walls or obstacles, would continuously accelerate. Thus, the convergence to a stationary state can only be explained by the non-trivial interaction between the flow and the curvature. Figure 2(b) shows the corresponding velocity streamlines in the stationary state, which are bent towards the center of the metric perturbation by the inertial forces. The attraction of the flow streamlines towards the bump can be explained by the attractive effect of the inertial forces for positive curvature. Accordingly, a negative curvature would exert repulsive inertial forces, repelling the flow streamlines from the bump. It is important to notice that the bending of the streamlines introduces velocity gradients ∇_*i*_*u*_*j*_ between adjacent fluid layers, which generate viscous stresses within the fluid. This mechanism also explains the convergence to the steady state, since the curvature-induced viscous forces oppose the pressure drop. At the same time, the presence of viscous stresses causes an irreversible dissipation of energy, which can be measured locally by the dissipation function *ψ* = (∇_*i*_*u*_*j*_)*σ*^*ij*^, as depicted in Fig. 2(b). The deviation of the velocity streamlines from the geodesic lines is explained by the fact that fluid flow – as the collective motion of interacting particles – behaves differently from non-interacting particles: While in curved spaces, non-interacting free particles always move along geodesics, the motion of the fluid is strongly influenced by the microscopic collisions, introducing momentum transfer between particles. Thus, the streamlines of a fluid deviate considerably from the geodesic lines in general.

In order to characterize the flow in terms of a Reynolds number, we define a characteristic length by the effective diameter of the metric perturbations, *d* = 2*r*_0_. Accordingly, the Reynolds number can be defined as *Re* = *d*Φ/*v* ≈ 0.6, where 
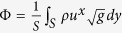
 denotes the mass flow, evaluated on an arbitrary channel cross-section *S*. The dependence of curvature-induced dissipation on the Reynolds number is two-fold, since the Reynolds number depends both on the flow velocity as well as on the viscosity of the fluid. Firstly, an increase of the flow velocity results in stronger inertial forces, inducing more shear flow and thus more dissipation. Secondly, the dissipation is proportional to the fluid viscosity, determining the strength of the viscous stresses (and thus the dissipation) within the flow.

### Curvature-induced dissipation in curved soap films

In order to estimate the significance of this effect in a real system, we performed a simulation of a 2D flowing soap film as considered for example in refs 13 and 14. In a real experiment, the soap film would be spanned between two nylon threads, and an out-of-plane curvature of the film may be created by a gentle jet of air, by an electrostatic field or by bending the confining nylon threads. In our simulations, the nylon threads are accounted for by using no-slip boundary conditions, and the curvature of the film is modeled by a Gaussian-shaped metric perturbation. Note that the nylon threads provide a significant additional source of dissipation, which dominates over the dissipation induced by curvature. Still, we can determine the relative magnitude of curvature-induced dissipation by comparing the dissipation in a flat soap film to the dissipation in a curved film, where curvature effects arise. To this end, we simulate a soap film of width *L* = 10 cm, height *h* = 1 *μ*m, mass density *ρ* = 10^−6^ g/cm^2^ and effective 2D dynamic viscosity *μ* = 10^−9^ Pa m s with a metric perturbation of range *r*_0_ = 1 cm. We find that the dissipation increases by about 1%, from 〈*ψ*_0_〉 = (6.348 ± 0.002) · 10^−8^ J/sm^2^ (flat film) to 〈*ψ*〉 = (6.392 ± 0.002) · 10^−8^ J/sm^2^ (curved film), due to curvature effects. This might seem a small correction, but it grows significantly with the number and strength of the metric perturbations, thus leading to non-negligible effects. For a system with 16 randomly arranged metric perturbations, for example, the dissipation increases to about 12%.

We would like to point out that in a real experiment with soap films, there might be additional effects influencing the flow, originating for example from the interaction between the soap film and the surrounding air (e.g. Ekman friction^16^, interfacial effects^17^,^18^) or from the variations in the film thickness. These effects can also contribute to the dissipation, but, for simplicity, are not taken into account in our simulations, which aim to provide a basic idea of the relative strength of curvature-induced dissipation.

### Transport law for flows in general geometries

While in a soap film experiment, the flow is strongly affected by the confining wires, adding a significant source of dissipation to the flow, we are numerically able to study curvature-induced dissipation in open systems without boundaries by using numerical simulations. Figure 3 depicts the flow through an open system with 16 randomly arranged metric perturbations, where the velocity streamlines are bent only by the intrinsic curvature. In order to find a general transport law, we have measured the total mass flow Φ for different values of the pressure gradient |∇*P*|. In all simulations, the flow converged to a stationary state by the curvature-induced viscous forces, which shows that the stationary state is a general feature of viscous flows in curved spaces. Figure 4 depicts the simulation results for Φ, measured in the stationary state, as function of |∇*P*|. As can be seen, for Φ ≲ 0.04, there is a linear correlation between |∇*P*| and Φ, where the flow is governed by the terms linear in the velocity in the Navier-Stokes equation (1). These terms represent curvature-induced viscous forces, 
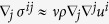
, originating from the viscous stress tensor and thus from the shear ∇_*j*_*u*^*i*^ induced between adjacent fluid layers. For stronger mass flows, Φ ≳ 0.04, the transport law in Fig. 4 requires a non-linear correction, originating from the terms quadratic in the velocity ~∇_*j*_(*ρu*^*i*^*u*^*j*^). In this regime, the quadratic terms 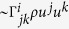 begin to dominate over the viscous forces, causing a break down of the linear law. An insightful quantity to characterize the stationary state is the friction factor *f* = |∇*P*|/Φ^2^*d*, expressing the ratio between the shear stress and the kinetic energy. The dependence of the friction factor on the Reynolds number *Re* = *d*Φ/*v* is depicted in the lower inset of Fig. 4. Finally, the upper inset of Fig. 4 depicts the dependence of the flux on the viscosity, measured at constant pressure drop, finding an anti-correlation between Φ and *v*. All these results can be summarized in the following transport law, describing both the linear regime and the non-linear effects:


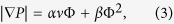


where *α* and *β* are parameters, which can be interpreted as reciprocal permeabilities, and *v* denotes the kinematic viscosity. Interestingly, Eq. (3) is similar to the Darcy-Forchheimer’s law for porous media^26^,^27^, where the dissipation originates from the interaction of the flow with solid obstacles. However, there are major physical differences regarding the underlying mechanisms in the two types of media. In a porous medium, the obstacles are impermeable solid structures, while the fluid motion is governed by the Navier-Stokes equation in flat space. In a curved medium, however, the background space itself is curved, inducing inertial forces, viscous stresses and thus dissipation. Furthermore, since the metric perturbations are permeable to the fluid, the flow typically does not form sharp channel-like paths as in a porous medium.

In order to formulate a transport law for general geometries, we study the dependence of the coefficients *α* and *β* on the parameters of the curved space. We have performed simulations for a wide range of metric parameters by varying the amplitude *a*_0_, the range *r*_0_ and the number density *n* = *N/V* of the metric perturbations, where *V* denotes the volume of the curved space. We observe that all the data collapse onto a single common curve when plotting *α* and *β* as function of the non-dimensionalized average metric perturbation 
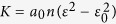
, where *ε* = *r*_0_/*λ* denotes the non-dimensionalized perturbation range, normalized by the characteristic distance between the perturbations, 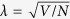, and *ε*_0_ ≈ 1.3. The resulting curves are depicted in Fig. 5, showing a linear increase of *α* and *β* with *K*. Accordingly, the permeabilities ~*α*^−1^, *β*^−1^ decrease with the average strength of the metric perturbation. This behavior agrees with our expectation, since the stronger the metric perturbations in the medium, the larger the effective resistance against the flow will be.

### Summary and outlook

Summarizing, we have presented a fundamental physical process inherent to fluid dynamics in curved space, namely curvature-induced dissipation. We have shown that local sources of curvature generate viscous stresses, which tend to narrow or widen the streamlines and thus introduce velocity gradients between adjacent fluid layers, leading to an irreversible dissipation of energy. In order to demonstrate the consequences of curvature-induced dissipation, we have studied media with randomly-distributed curvature sources. We have observed that the flow converges to a stationary equilibrium state solely due to curvature-induced dissipation. As a consequence, the flow has been found to satisfy a non-linear transport law, |∇*P*| = *αv*Φ + *β*Φ^2^, where the reciprocal permeabilities *α* and *β* are linearly correlated with the average metric perturbation in the medium.

The present work might have important implications on physical systems described by hydrodynamics on curved manifolds. For example, curvature-induced dissipation is expected to influence the two-dimensional fluidity and thus the biological function of curved lipid bilayers, constituting the cell membranes. In particular, the diffusive motion of the tracer particles used to measure the membrane viscosity^4^ is probably affected by the curvature. Furthermore, we expect analogous effects in relativistic flows described by a relativistic extension of the Navier-Stokes equations. For example, curvature-induced dissipation might influence astrophysical hydrodynamic systems, such as gases in stars or interstellar clouds, and might even play a role in controversial cosmological questions concerning e.g. the fluid dynamical description of dark matter and dark energy^28^,^29^. Another very interesting question is the relation of our work to theories of superfluidic films. In fact, the effect of curvature-induced dissipation in the form studied in this paper is not expected to arise in superfluids, since ideal superfluids, as considered for example by Turner *et al*. in ref. 30, possess zero viscosity in general, meaning that the dissipative mechanisms of conventional fluids are absent (even in curved spaces) and kinetic energy is thus totally conserved. Still, since inertial forces are also present in superfluids, we expect curvature to mainly cause deviations of the streamlines, resulting in shear flow, but not in energy dissipation. It would be interesting to explore these effects in a future investigation.

## Methods

### Lattice Boltzmann simulations

Flow through curved space is modeled by the lattice Boltzmann (LB) method on manifolds^22^,^23^,^24^, based on the LB equation


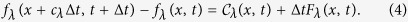


This equation describes the evolution of the distribution function *f*_*λ*_ in space and time according to kinetic theory on manifolds, characterized by the metric tensor *g*_*ij*_. All quantities are discretized on a uniform rectangular two-dimensional lattice, the *D*2*Q*17-lattice, consisting of 17 lattice vectors 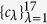 per node (see Suppl. Mat. for details). The lattice spacing is coupled to the LB time step by Δ*x* = Δ*y* = Δ*t*. While the left-hand side of the LB equation represents free streaming, the right-hand side accounts for collisions and inertial forces on the manifold. Collisions are described by the BGK collision operator 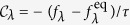
31, where *τ* is the relaxation time and 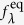 the Maxwell-Boltzmann equilibrium distribution in curved space. The inertial forces on the manifold enter the LB equation through the forcing term *F*_*λ*_, which needs special treatment in order to cancel spurious discrete lattice effects in the hydrodynamic equations (see Suppl. Mat. for details). In the hydrodynamic limit, the LB equation flows into the covariant Navier-Stokes conservation equations^25^,


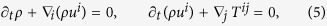


where the macroscopic fluid density *ρ* and fluid velocity 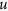 are obtained by taking the moments of the distribution function,


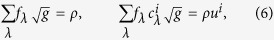


where 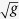 denotes the square root of the metric determinant, and the energy-stress tensor 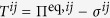 is composed of the free momenum-flux tensor Π^eq,*ij*^ and the viscous stress tensor *σ*^*ij*^:


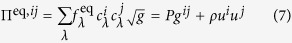



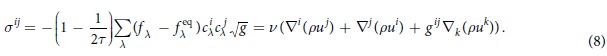


Here, *P* = *ρθ* denotes the hydrostatic pressure, *θ* the normalized temperature (we work in the isothermal limit *θ* = 1), *g*^*ij*^ the inverse metric tensor, 
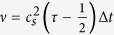
 the kinematic viscosity, and 
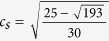
 the lattice specific speed of sound.

For the simulations performed for this paper, we use a time step of Δ*t* = 0.5 (corresponding to a grid size of 256 × 128 points) and set the fluid viscosity to 
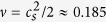
, which fixes the relaxation parameter *τ* accordingly. At the inlet and outlet, we use a non-equilibrium extrapolation method, ref. 32, to impose open boundaries with a pressure gradient, driving the fluid towards the outlet. No-slip boundaries (walls) are implemented by imposing zero flow velocity in the equilibrium distribution.

**Figure 1:**
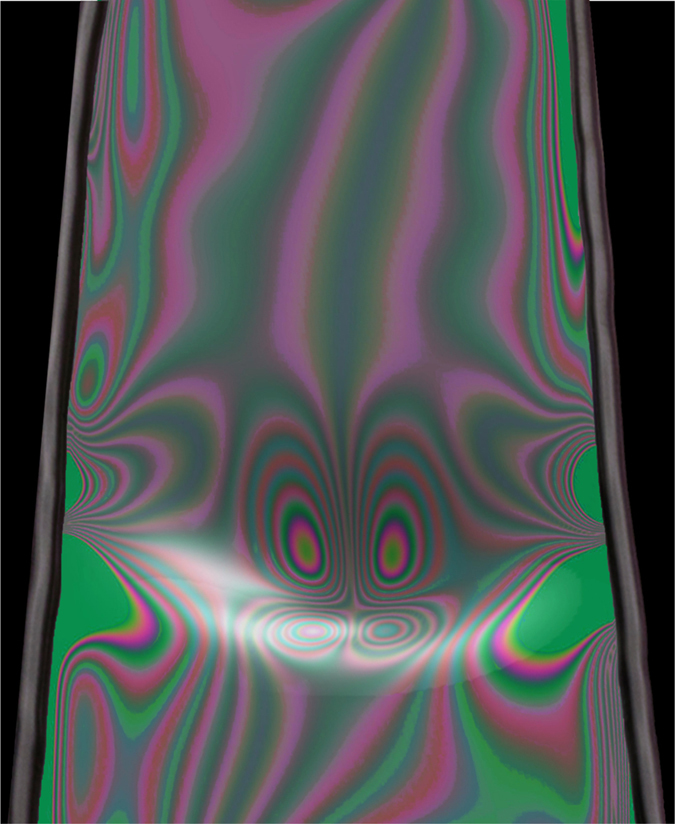
Numerical simulation of flow on a curved surface. The figure depicts a simulation of a 2D fluid film, spanned between two vertical wires and flowing downwards by gravity. In the center, the film possesses an out-of-plane curvature modeled by a Gaussian function. The colors represent the vorticity field of the flow, showing curvature-induced, quadrupol-shaped distortions next to the center.

**Figure 2:**
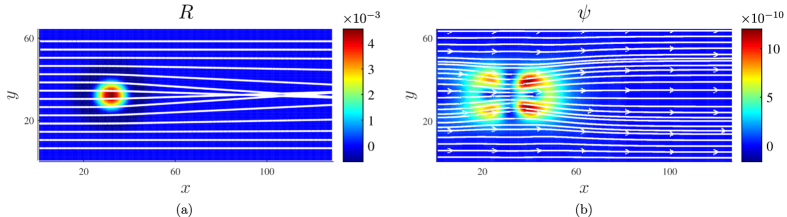
Simulation results for flow past a centered metric perturbation. The metric perturbation is of the shape 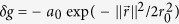 with amplitude *a*_0_ = −0.1 and width *r*_0_ = 6, embedded in a rectangular medium of size *L*_*x*_ × *L*_*y*_ = 128 × 64. The flow is driven by a pressure gradient of |∇*P*| = 5.8 × 10^−9^, and the kinematic viscosity of the fluid is set to *v* = 0.185 (all quantities are given in numerical units). At the inlet (*x* = 0) and outlet (*x* = *L*_*x*_), open boundary conditions have been applied, whereas periodic boundary conditions are used in *y*-direction in order to avoid dissipative effects originating from boundary walls. (**a**) The Ricci curvature scalar *R*, i.e. the strength of the curvature. The white lines represent the geodesic lines, which are calculated from the geodesic equation 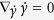. As can be seen, the convex curvature field exerts a focusing effect on the geodesics, caused by the attractive inertial force field. (**b**) The colors represent the local energy dissipation function 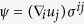 in the stationary state, where *σ*^*ij*^ denotes the viscous stress tensor. The dissipative effect around the curvature source is clearly visible, and the dissipation function shows a similar quadrupol pattern as the vorticity field in Fig. 1. The white lines represent the simulated flow streamlines, which are narrowed around the metric perturbation, thus inducing larger velocity gradients between adjacent fluid layers. The corresponding Reynolds number of the system is given by *Re* = 2*r*_0_Φ/*v* ≈ 0.6, where Φ denotes the mass flow.

**Figure 3:**
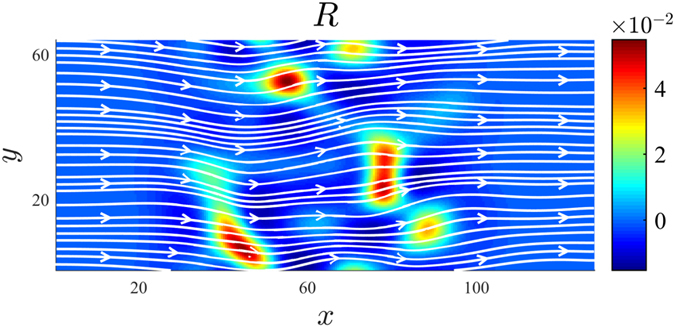
Flow past a randomly curved medium. The Ricci curvature scalar *R* for an open system with 16 randomly arranged, Gaussian-shaped metric perturbations of amplitude *a*_0_ = 0.1 and width *r*_0_ = 6. The colors illustrate the strength of the curvature, and the white lines represent the streamlines of the flow, driven by a pressure gradient of |∇*P*| = 5.8 × 10^−6^ with a kinematic viscosity of *v* = 0.185 (in numerical units). As can be seen, the flow streamlines are bent by the curvature.

**Figure 4:**
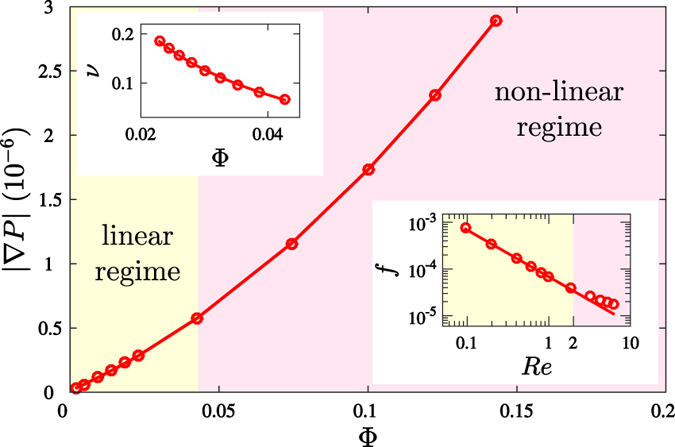
Transport law for a curved medium with 16 metric perturbations (amplitude *a*_0_ = 0.1, width *r*_0_ = 4). The solid line in the main plot represents a fit with respect to the non-linear transport law, |∇*P*| = *αv*Φ + *β*Φ^2^ for *α* = (5.8 ± 0.2) × 10^−5^ and *β* = (6.6 ± 0.3) × 10^−5^. *Upper inset:* Flux Φ as function of viscosity *v* at constant pressure drop |∇*P*| = 2.9 × 10^−7^. The solid line represents a fit using Eq. (3), obtaining *α* = (6.01 ± 0.06)10^−5^ and *β* = (6.7 ± 0.3) × 10^−5^, in agreement with the fitting values found above. *Lower inset:* Friction factor *f* = |∇*P*|/Φ^2^*d* as function of the Reynolds number *Re* = Φ*d/μ*. We observe a linear power law, *f* = *α/Re*, for small Reynolds numbers, *Re* ≲ 2, where the flow is dominated by viscous shear forces, while for higher Reynolds numbers, *Re* ≳ 2 the departure from the linear law is visible.

**Figure 5:**
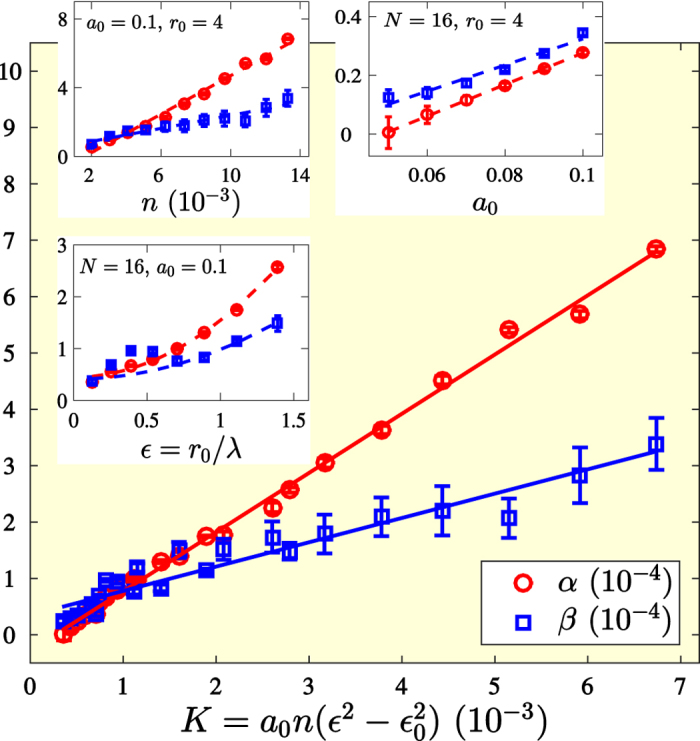
Dependence of the reciprocal permeabilities *α* and *β* on the geometry. Insets: *α* and *β* as function of the amplitude *a*_0_, dimensionless range *ε* = *r*_0_/*λ* and number density *n* = *N/V* of the metric perturbations. *Main plot:* Data collapse when plotting *α* and *β* as function of the dimensionless average metric perturbation 
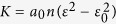
 for *ε*_0_ ≈ 1.3. As can be seen, *α* and *β* increase linearly with the average metric perturbation, and the solid lines represent linear fits (see Suppl. Mat. for the fitting coefficients). Consequently, the permeability of the medium decreases with increasing strength of the metric perturbation. In all plots, the error bars originate from fitting the non-linear transport law, |∇*P*| = *αv*Φ + *β*Φ^2^, to our data.

The simulation depicted in Fig. 1 is based on a grid of 512 × 128 lattice points, using open boundaries at the top and bottom of the channel, and no-slip boundary conditions at the walls. The curvature of the surface is modeled by a Gaussian function of amplitude *a*_0_ = 15 and width *r*_0_ = 28 (all quantities are given in numerical units). The flow is driven by a pressure gradient of ∇*P* = 9.2 × 10^−10^, and the vorticity field is measured with a finite difference method at time *t* = 1800. The colorbar is taken from ref. 33 and corresponds to the thickness-dependent colors of a soap film with refraction index *n* = 1.33, illuminated by white daylight (Illuminant D65).

In the soap film simulations, the average dissipation is calculated by


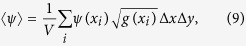


where the index *i* runs over all lattice points and 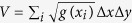 denotes the area of the curved film.

In order to prove that our simulation results are not biased by numerical errors, we have performed a finite resolution study, showing that in general our results are not appreciably affected by finite resolution effects (up to an error of about 1% for lattice spacing 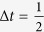). Regarding the dissipative effect of the curvature in curved soap films, we applied a finite resolution study (see Suppl. Mat.) to decrease the numerical error to about 0.03% by extrapolating the measurands towards increasing grid resolutions. This procedure was necessary since the effect of curvature, as compared to the dominating dissipative effect of the nylon threads, is relatively small and thus more sensitive to numerical errors. All other quantities measured in this paper are not affected by this issue, since they are less sensitive to numerical errors. We also have checked that our results are not constrained by the positions of the inlet and outlet. Details on the method validation can be found in the Suppl. Mat.

## Additional Information

**How to cite this article:** Debus, J.-D. *et al*. Energy dissipation in flows through curved spaces. *Sci. Rep.* **7**, 42350; doi: 10.1038/srep42350 (2017).

**Publisher's note:** Springer Nature remains neutral with regard to jurisdictional claims in published maps and institutional affiliations.

## Supplementary Material

Supplementary Information
