# Supplementary material for: Energy dissipation in flows through curved spaces
Source: Sci Rep. 2017 Feb 14;7:42350. doi: 10.1038/srep42350 (PMC5307957; doi:10.1038/srep42350)
Supplement: Supplementary Information [file srep42350-s1.pdf]

Energy dissipation in flows through curved spaces – Supplementary Information

J.-D. Debus[1,*], M. Mendoza[1], S. Succi[2], and H. J. Herrmann[1]

[1]ETH Zürich, Computational Physics for Engineering Materials, Institute for Building Materials, Wolfgang-Pauli-Str. 27, HIT, CH-8093 Zürich (Switzerland)
[2]Instituto per le Applicazioni del Calcolo C.N.R., Via dei Taurini, 19 00185, Rome (Italy)
[*]debusj@ethz.ch

The Method

In the following section, we prove that in the hydrodynamic limit, our curved-space lattice Boltzmann equation flows into the covariant Navier-Stokes equations with second order accuracy in space and time. To this end, we will use the following abbreviations for readability:

$$\overline{\sum}_\lambda := \sum_\lambda \sqrt{g} \quad , \quad D_t := \partial_t + c^i_\lambda \partial_i \quad , \quad \overline{\partial}_i := \partial_i - \Gamma^j_{ij}, \tag{1}$$

where $\sqrt{g}$ denotes the square root of the metric determinant, $\Gamma^i_{jk} = \frac{1}{2} g^{im}(\partial_j g_{km} + \partial_k g_{jm} - \partial_m g_{jk})$ the Christoffel symbols, and c^i_λ the lattice vectors.

Lattice Boltzmann model

The lattice Boltzmann equation in curved space is given by

$$f_\lambda(x + c_\lambda \Delta t, t + \Delta t) - f_\lambda(x,t) = \mathscr{C}_\lambda + \Delta t F_\lambda, \tag{2}$$

where the equilibrium distribution f^{eq}_λ as well as the forcing term F_λ are expanded into an orthonormal polynomial basis (tensor Hermite polynomials[1]) in order to recover their macroscopic moments exactly up to third order through Gauss-Hermite quadrature. Explicitly, the tensor Hermite polynomials are given by

$$\mathscr{H}_{(0)}(v) = 1, \quad \mathscr{H}^i_{(1)}(v) = v^i, \quad \mathscr{H}^{ij}_{(2)}(v) = v^i v^j - \delta^{ij}, \quad \mathscr{H}^{ijk}_{(3)}(v) = v^i v^j v^k - \left(\delta^{ij} v^k + \delta^{jk} v^i + \delta^{ki} v^j\right), \quad \dots \tag{3}$$

and satisfy the orthogonality relation up to third order (for the given third-order $D2Q17$-lattice):

$$\sum_\lambda w_\lambda \, \mathscr{H}^{I_n}_{(n),\lambda} \cdot \mathscr{H}^{J_m}_{(m),\lambda} = \delta_{nm} \delta^{I_n J_n}, \tag{4}$$

where $n,m = 0,1,2,3$ and $\mathscr{H}^{I_n}_{(n),\lambda} := \mathscr{H}^{I_n}_{(n)}(c_\lambda / c_s)$. Here, w_λ and c_λ are the lattice weights and velocities, respectively, (listed in Table 1), $c_s = \sqrt{\frac{25-\sqrt{193}}{30}}$ denotes the lattice specific speed of sound, $I_n = (i_1, \dots, i_n)$ and $J_m = (j_1, \dots, j_m)$ are index tuple and $\delta^{I_n J_n} := \sum_{\sigma \in S_n} (\delta^{i_1 j_{\sigma(1)}} \cdots \delta^{i_n j_{\sigma(n)}})$ denotes the fully symmetric tensorial Kronecker delta.

λ	c_λ	w_λ
1	$(0,0)$	$\frac{575+193\sqrt{193}}{8100}$
2-3<br>4-5	$(\pm1,0)$<br>$(0,\pm1)$	$\frac{3355-91\sqrt{193}}{18000}$
6-9	$(\pm1,\pm1)$	$\frac{655+17\sqrt{193}}{27000}$
10-13	$(\pm2,\pm2)$	$\frac{685-49\sqrt{193}}{54000}$
14-15<br>16-17	$(\pm3,0)$<br>$(0,\pm3)$	$\frac{1445-101\sqrt{193}}{162000}$

Table 1. Discrete velocity vectors c_λ of the D2Q17 lattice and the corresponding weights w_λ in the Hermite expansion.

The expanded equilibrium distribution reads

$$f_\lambda^{\mathrm{eq}}(x,t) = \frac{w_\lambda}{\sqrt{g}} \sum_{n=0}^{3} \frac{1}{n!\,c_s^n} a_{(n)}^{\mathrm{eq},I_n}(x,t)\,\mathscr{H}_{(n),\lambda}^{I_n}, \tag{5}$$

where the expansion coefficients are given by

$$a_{(0)}^{\mathrm{eq}} = \rho, \qquad a_{(1)}^{\mathrm{eq},i} = \rho u^i, \qquad a_{(2)}^{\mathrm{eq},ij} = \rho c_s^2 \Delta^{ij} + \rho u^i u^j, \qquad a_{(3)}^{\mathrm{eq},ijk} = \rho c_s^2 \left(\Delta^{ij} u^k + \Delta^{jk} u^i + \Delta^{ki} u^j\right) + \rho u^i u^j u^k, \tag{6}$$

where $\Delta^{ij} := g^{ij} - \delta^{ij}$. The corresponding moments read

$$\rho = \overline{\sum}_\lambda f_\lambda^{\mathrm{eq}}, \tag{7}$$

$$\rho u^i = \overline{\sum}_\lambda f_\lambda^{\mathrm{eq}} c_\lambda^i, \tag{8}$$

$$\Pi^{\mathrm{eq},ij} = \overline{\sum}_\lambda f_\lambda^{\mathrm{eq}} c_\lambda^i c_\lambda^j = \rho\left(c_s^2 g^{ij} + u^i u^j\right), \tag{9}$$

$$\Sigma^{\mathrm{eq},ijk} = \overline{\sum}_\lambda f_\lambda^{\mathrm{eq}} c_\lambda^i c_\lambda^j c_\lambda^k = \rho c_s^2\left(u^i g^{jk} + u^j g^{ik} + u^k g^{ij}\right) + \rho u^i u^j u^k. \tag{10}$$

The inertial forces on the manifold enter the LB equation through the forcing term F_λ, which needs special treatment in order to cancel spurious discrete lattice effects in the hydrodynamic equations (cf.[2]). To this end, we employ the trapezoidal rule for the time integration in the LB equation by using an improved forcing term:

$$F_\lambda(x,t) := \mathscr{F}_\lambda(x,t) + \frac{1}{2}\left(\mathscr{F}_\lambda(x + c_\lambda \Delta t, t) - \mathscr{F}_\lambda(x, t - \Delta t)\right), \tag{11}$$

where $\mathscr{F}_\lambda$ is expanded in Hermite polynomials,

$$\mathscr{F}_\lambda(x,t) = \frac{w_\lambda}{\sqrt{g}} \sum_{n=0}^{2} \frac{1}{n!\,c_s^n} b_{(n)}^{I_n}(x,t)\,\mathscr{H}_{(n),\lambda}^{I_n}, \tag{12}$$

with expansion coefficients

$$b_{(0)} = -\Gamma_{ij}^i \rho u^j - \Gamma_{ij}^j \rho u^i, \tag{13}$$

$$b_{(1)}^i = -\Gamma_{jk}^i T^{jk} - \Gamma_{jk}^j T^{ik} - \Gamma_{jk}^k T^{ji}, \tag{14}$$

$$b_{(2)}^{ij} = -\Gamma_{kl}^i \Sigma^{\mathrm{eq},jkl} - \Gamma_{kl}^j \Sigma^{\mathrm{eq},ikl} - \Gamma_{kl}^k \Sigma^{\mathrm{eq},ijl} - \Gamma_{kl}^l \Sigma^{\mathrm{eq},ijk} - c_s^2 \delta^{ij} b_{(0)}. \tag{15}$$

Here, $T^{ij} := \Pi^{\mathrm{eq},ij} - \sigma^{ij}$ represents the second order flux tensor, where

$$\sigma^{ij} = -\left(1 - \frac{1}{2\tau}\right) \overline{\sum}_\lambda \left(f_\lambda - f_\lambda^{\mathrm{eq}}\right) c_\lambda^i c_\lambda^j, \tag{16}$$

denotes the viscous stress tensor. The corresponding moments of the forcing term are given by

$$A = \overline{\sum}_\lambda \mathscr{F}_\lambda = -\Gamma_{ij}^i \rho u^j - \Gamma_{ij}^j \rho u^i, \tag{17}$$

$$B^i = \overline{\sum}_\lambda \mathscr{F}_\lambda c_\lambda^i = -\Gamma_{jk}^i T^{jk} - \Gamma_{jk}^j T^{ik} - \Gamma_{jk}^k T^{ji}, \tag{18}$$

$$C^{ij} = \overline{\sum}_\lambda \mathscr{F}_\lambda c_\lambda^i c_\lambda^j = -\Gamma_{kl}^i \Sigma^{\mathrm{eq},jkl} - \Gamma_{kl}^j \Sigma^{\mathrm{eq},ikl} - \Gamma_{kl}^k \Sigma^{\mathrm{eq},ijl} - \Gamma_{kl}^l \Sigma^{\mathrm{eq},ijk}. \tag{19}$$

With the improved forcing term, the Navier-Stokes equations are recovered at second order in space and time, as will be shown in the following section. This significantly improves the accuracy of the results. The advantage of this force correction scheme, as compared to the commonly used scheme by Guo *et al.*[2], is that the macroscopic moments (density ρ, velocity u etc.) are not modified and thus retain their original physical meaning. Moreover, for the special forcing term used in our model, a modification of the macroscopic moments according to Ref.[2] would require solving a complicated system of equations for ρ and u. With our method, such modifications of the macroscopic moments are dispensed by employing the trapezoidal rule for the time integration in the lattice Boltzmann equation.

Chapman-Enskog expansion

In order to prove that our model recovers the correct hydrodynamic conservation equations (covariant Navier-Stokes equations), we perform a Chapman-Enskog multiscale expansion by expanding the distribution function as well as the time and space derivatives in terms of the Knudsen number ε[3,4]:

$$
\begin{aligned}
& f = f^{(0)} + \varepsilon f^{(1)} + \varepsilon^2 f^{(2)} + \ldots, \\
& \partial_t = \varepsilon \partial_t^{(1)} + \varepsilon^2 \partial_t^{(2)} + \ldots, \\
& (\partial_i, \mathscr{F}, A, B^i, C^{ij}) = \varepsilon(\partial_i^{(1)}, \mathscr{F}^{(1)}, A^{(1)}, B^{(1),i}, C^{(1),ij})
\end{aligned}
$$

Plugging everything into Eq. (2) and comparing orders of ε, we obtain the following equations:

$$\mathscr{O}(\varepsilon^0): \quad f_\lambda^{(0)} = f_\lambda^{\mathrm{eq}}, \tag{20}$$

$$\mathscr{O}(\varepsilon^1): \quad D_t^{(1)} f_\lambda^{(0)} = -\frac{1}{\tau \Delta t} f_\lambda^{(1)} + \mathscr{F}_\lambda^{(1)}, \tag{21}$$

$$\mathscr{O}(\varepsilon^2): \quad \partial_t^{(2)} f_\lambda^{(0)} + \left(1 - \frac{1}{2\tau}\right) D_t^{(1)} f_\lambda^{(1)} = -\frac{1}{\tau \Delta t} f_\lambda^{(2)}. \tag{22}$$

Moments of Eq. (21-22)

Taking the moments of Eq. (21) yields:

$$\sum_\lambda (21): \quad \partial_t^{(1)} \rho + \overline{\partial}_i^{(1)} \left(\rho u^i\right) = A^{(1)}, \tag{23}$$

$$\sum_\lambda c_\lambda^i \,(21): \quad \partial_t^{(1)} \left(\rho u^i\right) + \overline{\partial}_j^{(1)} \Pi^{(0),ij} = B^{(1),i}, \tag{24}$$

where $\Pi^{(0),ij} = \Pi^{\mathrm{eq},ij} = \rho \left(c_s^2 g^{ij} + u^i u^j\right)$, and the $\overline{\partial}_i$ derivative originates from

$$\sum_\lambda c_\lambda^i \partial_i f_\lambda^{(0)} = \partial_i \sum_\lambda \sqrt{g}\, c_\lambda^i f_\lambda^{(0)} - \sum_\lambda (\partial_i \sqrt{g})\, c_\lambda^i f_\lambda^{(0)} = \partial_i(\rho u^i) - \Gamma_{ij}^j (\rho u^i) =: \overline{\partial}_i (\rho u^i), \tag{25}$$

where we have used the identity $\partial_i \sqrt{g} = \Gamma_{ij}^j \sqrt{g}$. The moments of Eq. (22) are given by

$$\sum_\lambda (22): \quad \partial_t^{(2)} \rho = 0, \tag{26}$$

$$\sum_\lambda c_\lambda^i \,(22): \quad \partial_t^{(2)} \left(\rho u^i\right) = \overline{\partial}_j^{(1)} \sigma^{(1),ij}, \tag{27}$$

where $\sigma^{(1),ij}$, the viscous stress tensor (rescaled by ε), is defined as

$$\sigma^{(1),ij} = -\left(1 - \frac{1}{2\tau}\right) \sum_\lambda c_\lambda^i c_\lambda^j f_\lambda^{(1)}. \tag{28}$$

The explicit expression for the viscous stress tensor will be derived later.

Continuity Equation

For the continuity equation, we add $\varepsilon \cdot (23)$ and $\varepsilon^2 \cdot (26)$:

$$\partial_t \rho + \overline{\partial}_i \left(\rho u^i\right) = A \tag{29}$$

After inserting the explicit expression for A (17), we obtain the correct continuity equation:

$$\partial_t \rho + \nabla_i \left(\rho u^i\right) = 0, \tag{30}$$

where ∇ denotes the covariant derivative.

Momentum Equation

Adding $\varepsilon \cdot (24)$ and $\varepsilon^2 \cdot (27)$ yields the momentum conservation equation:

$$\partial_t \left(\rho u^i\right) + \overline{\partial}_j \Pi^{(0),ij} = \overline{\partial}_j \sigma^{ij} + B^i. \tag{31}$$

Inserting the explicit expression for B^i (18) and $\Pi^{(0),ij} = \Pi^{\mathrm{eq},ij}$ (9) yields the familiar Navier-Stokes equation:

$$\partial_t \left(\rho\, u^i\right) + \nabla_j \left(\rho\, u^i u^j\right) = -\nabla^i P + \nabla_j \sigma^{ij}, \tag{32}$$

where $P = \rho\, c_s^2$ denotes the hydrostatic pressure.

Viscous Stress Tensor

For the derivation of the viscous stress tensor σ^{ij}, we rewrite

$$\sigma^{ij} = -\left(1-\frac{1}{2\tau}\right)\varepsilon\overline{\sum}_\lambda c^i_\lambda c^j_\lambda f^{(1)}_\lambda \overset{(21)}{=} \left(\tau-\frac{1}{2}\right)\Delta t\,\varepsilon\overline{\sum}_\lambda c^i_\lambda c^j_\lambda\left(D^{(1)}_t f^{(0)}_\lambda - \mathscr{F}^{(1)}_\lambda\right) = \left(\tau-\frac{1}{2}\right)\Delta t\left(\overline{\partial}_k\Sigma^{\mathrm{eq},ijk} - C^{ij}\right), \tag{33}$$

where we have assumed that $\left(\tau-\frac{1}{2}\right)\Delta t\,\partial^{(1)}_t\Pi^{(0),ij} \ll 1$. After plugging in the explicit expressions for $\Sigma^{\mathrm{eq},ijk}$ (10) and C^{ij} (19), we obtain the familiar expression for the viscous stress tensor in the incompressible limit:

$$\sigma^{ij} = \nu\left(\nabla^i(\rho u^j) + \nabla^j(\rho u^i) + g^{ij}\nabla_k(\rho u^k)\right), \tag{34}$$

where we have defined $\nu := \left(\tau-\frac{1}{2}\right)\Delta t\,c_s^2$ and neglected terms of the order $\mathscr{O}(u^3)\sim\mathscr{O}(\mathrm{Ma}^3)$, Ma being the Mach number.

Finite resolution study

In order to proof the physicality of our lattice Boltzmann simulations, we have performed a finite resolution study. To this end, we have measured the transport law, $|\nabla P| = \alpha\nu\Phi + \beta\Phi^2$, for a curved medium with 16 metric perturbations ($a_0 = 0.1$, $r_0 = 4$) for different grid resolutions Δ^{-1}, as depicted in Fig. 1. As can be seen, the distance between the curves decreases

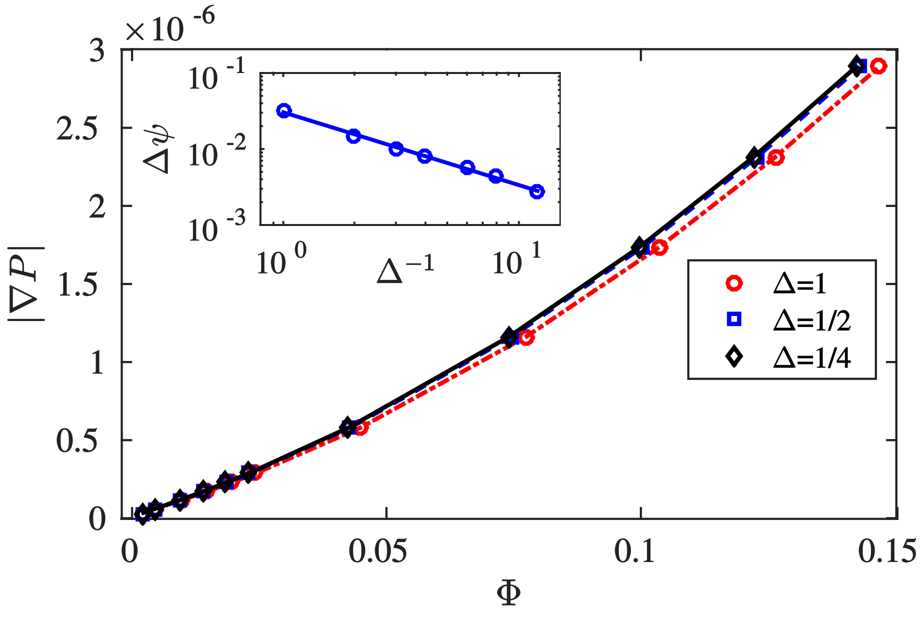

Figure 1. Transport law for a curved medium with 16 metric perturbations ($a_0 = 0.1$, $r_0 = 4$) for different discretization steps Δ. *Inset:* Relative error of the total dissipation function, $\Delta\psi$, as function of the grid resolution Δ^{-1} for a single metric perturbation ($a_0 = -0.1$, $r_0 = 6$).

rapidly with increasing resolution, which can be quantified by the relative L^2-difference, being less than 1% between the dashed ($\Delta = \frac{1}{2}$) and solid ($\Delta = \frac{1}{4}$) curve. Furthermore, we measured the finite resolution effects on the average dissipation, given by $\langle\psi\rangle = \frac{1}{V}\sum_i \psi(x_i)\sqrt{g(x_i)}\Delta x\Delta y$, where i runs over all lattice points and $V = \sum_i\sqrt{g(x_i)}\Delta x\Delta y$. In the inset of Fig. 1, the relative error $\Delta\psi = |\langle\psi\rangle - \langle\psi\rangle_\infty|/\langle\psi\rangle_\infty$ is depicted as function of the resolution Δ^{-1} for a single metric perturbation of amplitude $a_0 = -0.1$ and width $r_0 = 6$ at time $t = 10^5$. Here, $\langle\psi\rangle_\infty = (2.991\pm0.002)\times10^{-9}$ has been determined by extrapolation towards infinite grid resolutions. As can be seen, the error follows a power law $\Delta\psi\sim\Delta^\gamma = \Delta x^\gamma = \Delta y^\gamma = \Delta t^\gamma$, where γ characterizes the convergence of the LB method with the integration time step Δt and the lattice spacings Δx, Δy. In particular, the error decreases rapidly with the grid resolution, falling below 1% for $\Delta^{-1}\geq 2$. Altogether, this shows that our simulation results are not appreciably affected by finite resolution effects (up to an error of about 1% for $\Delta = \frac{1}{2}$).

While a numerical error of 1% is sufficiently low in general, there are particular measurands which require higher precision, for example the relative increase in dissipation depicted in Section "Curvature-induced dissipation in curved soap films", which

itself is of order 1%. In this case, we have applied a finite resolution study by systematically increasing the grid resolution and extrapolating the measurand towards an infinite resolution, resulting in a significantly lower error of about 0.03%.

We also have checked that our simulation results are not constrained by the positions of the inlet and outlet. To this end, we have performed additional simulations, putting inlet and outlet twice as far from the metric perturbations, observing that our simulation results do not change with the inlet and outlet positions (if they are sufficiently apart as in all the simulations presented in the paper).

Permeability fitting functions

Table 2 contains the fitting functions corresponding to the permeability plots in Fig. 5 in the Letter.

$\alpha(n)$	$=$	$(5.6 \pm 0.5) \times 10^{-2} n - (8.9 \pm 3.9) \times 10^{-5}$
$\beta(n)$	$=$	$(1.9 \pm 0.4) \times 10^{-2} n - (5.0 \pm 3.8) \times 10^{-5}$
$\alpha(a_0)$	$=$	$(1.07 \pm 0.04) \times 10^{-3} a_0 - (5.2 \pm 0.3) \times 10^{-5}$
$\beta(a_0)$	$=$	$(8.9 \pm 2.6) \times 10^{-4} a_0 - (2.4 \pm 2.0) \times 10^{-5}$
$\alpha(\varepsilon)$	$=$	$(1.1 \pm 0.8) \times 10^{-4} \varepsilon^2 - (4.5 \pm 0.7) \times 10^{-5}$
$\beta(\varepsilon)$	$=$	$(5.7 \pm 1.3) \times 10^{-5} \varepsilon^2 - (4.1 \pm 1.4) \times 10^{-5}$
$\alpha(K)$	$=$	$(1.05 \pm 0.03) \times 10^{-1} K - (2.8 \pm 0.8) \times 10^{-5}$
$\beta(K)$	$=$	$(4.3 \pm 0.6) \times 10^{-2} K - (3.5 \pm 1.6) \times 10^{-5}$

Table 2. Fitting functions for the reciprocal permeabilities α and β.

References

1. Grad, H. Note on n-dimensional hermite polynomials. *Communications on Pure and Applied Mathematics* **2**, 325–330 (1949).

2. Guo, Z., Zheng, C. & Shi, B. Discrete lattice effects on the forcing term in the lattice boltzmann method. *Phys. Rev. E* **65**, 046308 (2002).

3. Wolf-Gladrow, D. A. *Lattice-gas cellular automata and lattice Boltzmann models: an introduction* (Springer Science & Business Media, 2000).

4. Buick, J. M. & Greated, C. A. Gravity in a lattice boltzmann model. *Phys. Rev. E* **61**, 5307–5320 (2000).

Acknowledgements

We acknowledge financial support from the European Research Council (ERC) Advanced Grant 319968-FlowCCS.
